# The Poor-Law Medical Officers' Association

**Published:** 1914-06-06

**Authors:** 


### THE POOR-LAW MEDICAL OFFICERS' ASSOCIATION.

At a recent meeting of the Council of the Poor-Law Medical Officers' Association of England and Wales the annual report was considered and adopted. The report shows that much useful and valuable work has been carried out during the past year. Action has been taken or advice given to medical officers in connection with disputes in many Poor-Law Unions, with the result that in most cases the dispute has been settled to the satisfaction of all concerned. The value of the Association to the individual Poor-Law medical officer can hardly be over-estimated. The collective advice of the experienced and expert members of the Council is at his disposal should any difficulty arise between him and his Board, and if the dispute has to be carried to the Local Government Board for decision he has the advantage of having his case stated for him by men accustomed to such work who can set out the facts in the best way and collate them with the conditions and experience of other Unions.

At the same meeting the Council adopted a report on the Poor-Law Institutions Order. The report agrees in general with the criticisms we have published on the Order in our columns. It protests against the increased work thrown upon the medical officer without any provision for increase in his remuneration; against the waste of time involved in the frequent periodical examinations of healthy children in the manner prescribed in the Order; and against the subordination of the medical officer to the workhouse master. In this connection the report says: "The Council is quite unable to approve of any system that places the control of the sick wards in the hands of a non-medical officer. To subordinate the medical and nursing staff to such an official is, in its opinion, radically wrong, and should not be permitted. The Council receives many complaints from workhouse medical officers that workhouse masters use the powers given to them in a manner detrimental to the interests of the sick, and that the subordinate position of the medical officer often renders it impossible for him to obtain that prompt obedience to his orders which is essential. The Council is of opinion that, unless both the status and the remuneration of the workhouse medical officer are improved, the existing difficulty of obtaining such officers will be very considerably increased." With these criticisms we are in entire accord.

Any medical man who contemplates applying for the post of medical officer to a non-separated workhouse infirmary should clearly understand that the master is the administrative head of the sick wards, that the medical officer is his subordinate, and that one of the duties of the master specifically laid down in the Poor-Law Institutions Order is "to take care that all sick and insane inmates and infants are duly visited by the medical officer." Where the master is well educated, cultured, and sympathetic, or where, without these qualifications, he recognises that his nominal inferior must be supreme in the sick wards, no difficulties may arise. But it is notorious that in many workhouses neither of these conditions obtains, and the position of the medical officer is then likely to be intolerable. Three instances are given in the Council's reports showing how the Order ignores the resident medical officer even in the most elementary matters of administration. An inmate of a sick ward who wishes to discharge himself is required to give notice of his intention to do so to the master; the midwife, if she requires medical assistance, must apply to the master to send for the medical officer; and, finally, the master is directed, without any consultation with the medical officer, to send out notices to the friends if he imagines a patient to be dangerously ill. Medical men will be well advised if they abstain from applying for a resident post at a non-separated workhouse infirmary until these anomalies are rectified and the administration of the sick wards is placed entirely in the hands of the medical officer, with the master in his proper position as their steward.

